# Supplementary material for: Gastroesophageal reflux disease symptoms and risk of atrial fibrillation in a population-based cohort study (the HUNT study)
Source: PLoS One. 2024 May 31;19(5):e0304624. doi: 10.1371/journal.pone.0304624 (PMC11142718; doi:10.1371/journal.pone.0304624)
Supplement: S1 Table — (DOCX) [file pone.0304624.s001.docx]

| Supplemental Table 1. Characteristics of in- and excluded study population | | | | |
| --- | --- | --- | --- | --- |
|  | Included  n= 34,120 | | Excluded  n= 16,684 | |
| Female, n (%) | 19,105 (55.9) |  | 8,650 (52.1) |  |
| Age at Baseline, years (SD) | 52.8 (14.8) |  | 53.9 (18.4) |  |
| BMI (kg/m^2^) (SD) | 27.2 (4.4) |  | 27.2 (4.5) |  |
| Hypertension, n (%) | 13,242 (38.8) |  | 7,120 (42.7) |  |
| Diabetes mellitus, n (%) | 1,521 (4.5) |  | 920 (5.5) |  |
| C-reactive protein (mg/l) (IQR) | 2.6 (2.0) |  | 3.0 (2.4) |  |
| High-density lipoprotein (mmol/l) (SD) | 1.4 (0.4) |  | 1.3 (0.4) |  |
| Total cholesterol (mmol/l) (SD) | 5.5 (1.1) |  | 5.4 (1.1) |  |
| COPD, n (%) | 1,089 (3.2) |  | 655 (3.9) |  |
| Angina pectoris, n (%) | 1,084 (3.2) |  | 914 (5.5) |  |
| Myocardial infarction, n (%) | 943 (2.8) |  | 759 (4.6) |  |
| Alcohol consumption |  |  |  |  |
| Abstainers, n (%) | 6,671 (19.6) |  | 3,605 (21.6) |  |
| Light drinkers, n (%) | 20,693 (60.7) |  | 8,745 (52.4) |  |
| Moderate drinkers, n (%) | 6,270 (18.4) |  | 2,920 (17.5) |  |
| Heavy drinkers, n (%) | 486 (1.4) |  | 232 (1.4) |  |
| Smoking status |  |  |  |  |
| Never, n (%) | 14,696 (43.1) |  | 6,357 (38.1) |  |
| Former, n (%) | 11,429 (33.5) |  | 4,685 (28.1) |  |
| Occasionally, n (%) | 2,372 (7.0) |  | 1,251 (7.5) |  |
| Current, n (%) | 5,623 (16.5) |  | 2,962 (17.8) |  |
| Physical inactivity |  |  |  |  |
| Inactive, n (%) | 6,919 (20.3) |  | 4,050 (24.3) |  |
| Active, n (%) | 27,201 (79.7) |  | 11,701 (70.1) |  |
| Education |  |  |  |  |
| Lower secondary, n (%) | 7,414 (21.7) |  | 4,516 (27.1) |  |
| Upper secondary, n (%) | 16,930 (49.6) |  | 7,670 (46.0) |  |
| Tertiary, n (%) | 9,776 (28.7) |  | 3,333 (20.0) |  |
| Marital Status |  |  |  |  |
| Single, n (%) | 7,320 (21.5) |  | 4,433 (26.6) |  |
| Married, Cohabitant, n (%) | 21,019 (61.6) |  | 8,717 (52.3) |  |
| Widow, Divorced, Separated, n (%) | 5,781 (16.9) |  | 3,464 (20.8) |  |
| Values are presented as mean ± standard deviation for normal distributed continuous variables, mean and interquartile range (IQR) for not normal distributed continuous variables and number (percentages). BMI body mass index. COPD chronic obstructive lung disease. n= number. | | | | |
